# Simultaneously measuring multiple protein interactions and their correlations in a cell by Protein-interactome Footprinting

**DOI:** 10.1038/srep45169

**Published:** 2017-03-24

**Authors:** Si-Wei Luo, Zhi Liang, Jia-Rui Wu

**Affiliations:** 1 Key Laboratory of Systems Biology, Institute of Biochemistry and Cell Biology, Shanghai Institutes for Biological Sciences, Chinese Academy of Sciences, Shanghai, China; 2 Hefei National Laboratory for Physical Sciences at Microscale and School of Life Sciences, University of Science & Technology of China, Hefei, China; 3 School of Life Science and Technology, ShanghaiTech University, Shanghai, China

## Abstract

Quantitatively detecting correlations of multiple protein-protein interactions (PPIs) *in vivo* is a big challenge. Here we introduce a novel method, termed Protein-interactome Footprinting (PiF), to simultaneously measure multiple PPIs in one cell. The principle of PiF is that each target physical PPI in the interactome is simultaneously transcoded into a specific DNA sequence based on dimerization of the target proteins fused with DNA-binding domains. The interaction intensity of each target protein is quantified as the copy number of the specific DNA sequences bound by each fusion protein dimers. Using PiF, we quantitatively reveal dynamic patterns of PPIs and their correlation network in *E. coli* two-component systems.

In general, a human cell might contain about 130,000 binary PPIs^1^, and over 80% of proteins operate in complexes^2^. Since physical PPIs among these proteins could affect each other, the influences between different PPIs may act a very important role in protein interactome. To quantify the correlation of different PPIs, simultaneous and quantitative detection of multiple PPIs in a cell is needed. So far, there is no method available for directly measuring such system property of protein interactome.

Based on yeast two-hybrid (Y2H), the high-throughput Y2H approach has been applied for genome-wide screen of physical PPIs in human cells^3^. However, this assay mainly provides qualitative data and could not simultaneously detect multiple physical PPIs in a cell. Based on protein-fragment complementation assay, other binary PPI detecting approaches such as bimolecular fluorescence complementation (BiFC) assay^4^, luminescence-based mammalian interactome mapping (LUMIER)^5^ and dual luminescence-based co-immunoprecipitation (DULIP) assay^6^ have been developed (reviewed by ref. 7). Although these methods could be used for large-scale interaction screens, they hardly detect multiple physical PPIs in one cell quantitatively and simultaneously. By using these methods, the intensity of a physical PPI under the influences of other PPIs or the correlations of different physical PPIs can not be measured.

To address these challenges, we developed a novel method, called Protein-interactome Footprinting (PiF), for the simultaneous and quantitative measurement of multiple physical PPIs of interactome in a cell by directly transcoding each physical PPI signal into a specific DNA sequence. By using this method, we observed the correlations of PPIs in *E. coli* two-component systems (TCS), and quantified them as PPI correlation network (PPICN).

## Results

### Design concept and workflow of Protein-interactome Footprinting

Based on the principle of transcoding signal from a PPI into a specific DNA sequence^8^, we needed to solve following two problems to invent a method to achieve measuring multiple PPI in one cell simultaneously: (1) design of orthogonal pairs of DNA binding domains and specific DNA sequences; (2) measure the copy numbers of these different DNA sequences at the same time.

**Figure 1:**
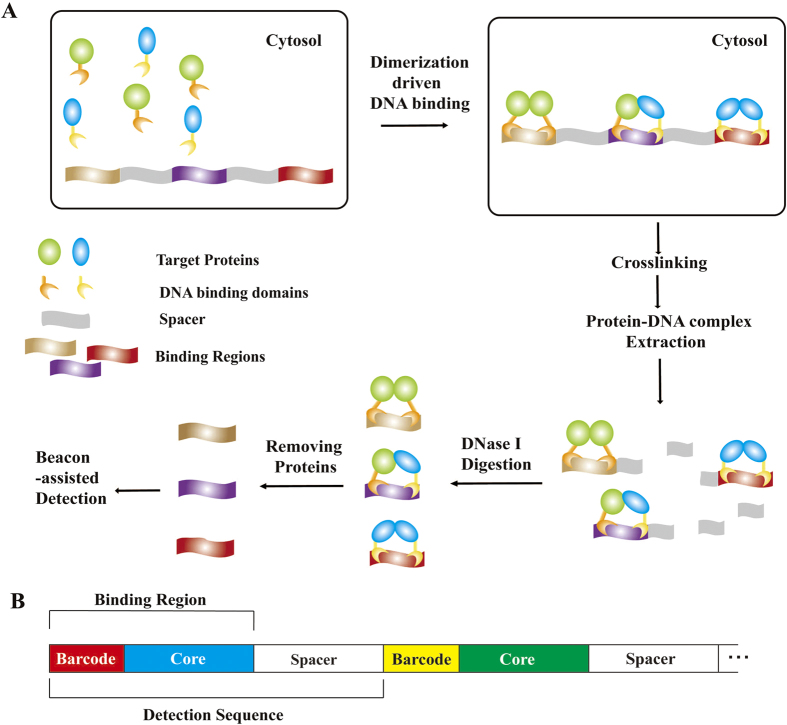
Principle of Protein interactome footprinting (PiF) method. (**A**) Workflow of the PiF method. The simultaneous detection ability of PiF include: different PPI driven DNA binding processes happen simultaneously in a cell, and copy numbers of different detection DNA are measured simultaneously by BAD assay. (**B**) The composition of the DNA sequences for detection. There are two sectors in these DNA sequences for detection, one is binding region (BR) composed by specific core sequence and barcode sequence, the other is spacer sequence for separating different BRs.

According to this concept, we worked out the simplest detection-system for multiple physical PPIs, i.e. simultaneously detecting three different PPIs between two different proteins in one cell (Fig. 1A), called Protein-interactome Footprinting (PiF), as follows (Fig. 1A): (1) each target protein is fused with a special DNA binding domain, which could form three different fusion protein dimers; (2) different fusion protein dimers recognize different specific DNA sequences and protect them from DNaseI digestion, and there are two sectors in these DNA sequences for detection (Fig. 1B), one is binding region (BR) composed by specific core sequence and barcode sequence, the other is spacer sequence for separating different BRs; (3) each physical PPI is transcoded into a specific DNA sequence through this process, and the copy numbers of these different DNA sequences are simultaneously measured by beacon-assisted detection (BAD)^9^.

### Design and selection of specific core sequences

In this study, We generated two different DNA binding domains CI(N, wt) and CI(N, mut) (Table S1) to fuse with two different target proteins, which enables the discrimination of the three possible binary interactions via CI(N,wt)/CI(N,wt), CI(N,wt)/CI(N,mut) and CI(N,mut)/CI(N,mut) DNA binding domain combinations.

**Figure 2:**
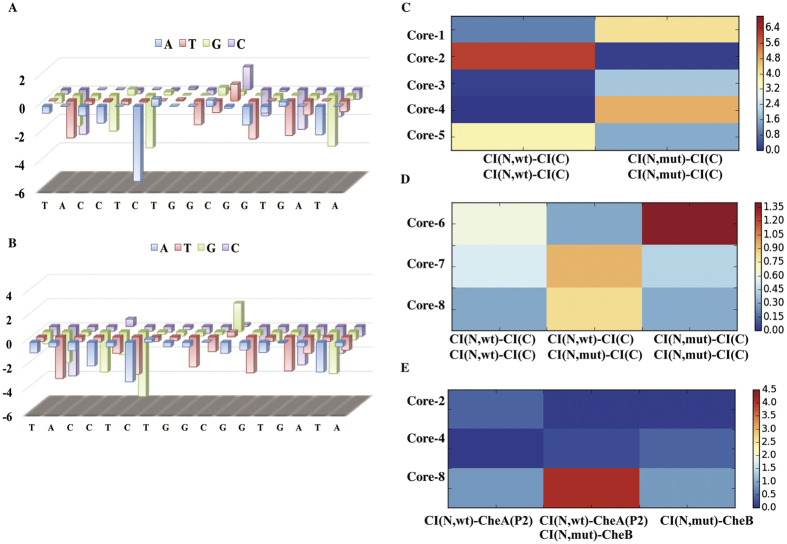
Design and selection of core sequences. (**A**) Interaction energy changes of CI(N,wt) homodimer-DNA complex with different DNA single-point mutations. (**B**) Interaction energy changes of CI(N,wt)CI(N,mut) heterodimer-DNA complex with different DNA single-point mutations. (**C**) Normalized PdF signals of transcoding CI(C) dimerization to core sequences (core-1~5) through CI(N,wt) or CI(N,mut) homodimer. (**D**) Normalized PdF signals of transcoding CI(C) dimerization to core sequences (core-6~8) through CI(N,wt) homodimer, CI(N,mut) homodimer or CI(N,wt)CI(N,mut) heterodimer. (**E**) Normalized PdF signals of transcoding CheA(P2)-CheB interaction to core sequences (core-2, core-4, core-8) through CI(N,wt)CI(N,mut) heterodimer, where fusion proteins CI(N,wt)-CheA(P2) and CI(N,mut)-CheB are not able to form homodimer.

To recognize these different PPIs derived combinations, we needed to design specific core sequences. By using FoldX^10^, we built the complex structure of CI(N,wt)/CI(N,mut) heterodimer binding with DNA, which is based on the complex structure of CI(N,wt)/CI(N,wt) homodimer binding with DNA (PDB:1LMB), then structures corresponding to each of the single point mutants of DNA sequences in these two complexes were generated. The interaction energy changes of CI(N,wt)/CI(N,wt)–DNA complex (Fig. 2A) and CI(N,wt)/CI(N,mut)–DNA complex (Fig. 2B) were obtained by comparing the interaction energies of DNA mutant structures from each complex with each wild type.

According to these calculations, we designed five core sequences (core-1~5) for CI(N,wt)/CI(N,wt) and CI(N,mut)/CI(N,mut), three core sequences (core-6~8) for CI(N,wt)/CI(N,mut) (Table S1). To validate their specificity in PPI signal transcoding, we measured PdF signals^8^ of these core sequences binding with different DNA binding domain combinations (Fig. 2C–E). From these results, three orthogonal combinations of DNA binding domains and the corresponding core sequences were identified, including CI(N,wt)/CI(N,wt) + core-2, CI(N,mut)/CI(N,mut) + core-4, and CI(N,wt)/CI(N, mut) + core-8.

### Design and validation of barcode sequences and molecular beacons

**Figure 3:**
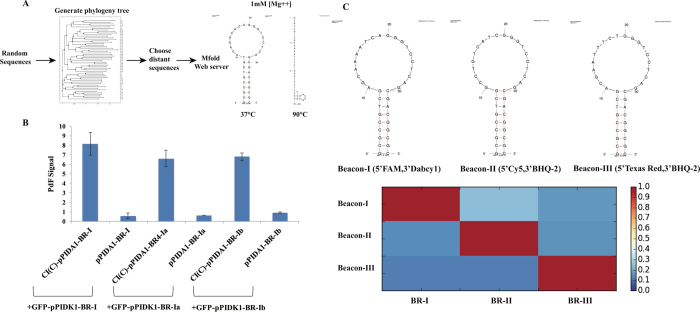
Design and validation of barcode sequences and molecular beacons. (**A**) Process of barcode sequences and molecular beacons design. The barcode sequences were inserted into GCGCCGTCG**(…)** GGGTCCTCAGCGACGGCGC as beacons. According to the prediction of Mfold web server, the proper folding conformation of beacon in1 mM [Mg^++^], at 37 °C is stem-loop with the barcode sequence in the loop, and the proper folding conformation of beacon in1 mM [Mg^++^], at 90 °C is basically linear. (**B**) The PdF signals of CI(N,wt)-CI(C) dimer and CI(N,wt)-GFP monomer measured by using core-1 sequence linked with different barcode sequences. In this experiment, we constructed two dual-vector system, CI(C)-pPIDA1 + GFP-pPIDK1 for CI(N,wt)-CI(C) dimerization as a positive control and pPIDA1 + GFP-pPIDK1 for CI(N,wt)-GFP monomer as a negative control (Error bars indicate SD of PdF signals from three independent biological samples). (**C**) Three different beacons designed for PiF assay. Each beacon has a stem-loop structure with modification of different fluorophore-quencher pairs. The heatmap gives the specificity of the designed beacons to different BR sequences; red color represents high specificity and blue color represents low specificity.

We designed the barcode sequences linked to core sequences to mark above three different core sequences in BAD. As the process shown in Fig. 3A, 10 nt length random sequences were generated, and the phylogeny tree of these random sequences were drawn by using Clustal Omega web server. Then, we chose three distant sequences as candidate barcodes from the phylogeny tree to insert into the sequence GCGCCGTCG**(…)** GGGTCCTCAGCGACGGCGC as molecular beacons and used Mfold^11^ web server to validate their folding conformations. Finally, we selected three specific barcode (bar-1, bar-2 and bar-3, Table S1) for each of three core sequences, and obtained three BRs (BR-I = bar-1 + core-2, BR-II = bar-2 + core-4 and BR-III = bar-3 + core-8, Table S1) for PiF method by linking core sequences with their specific barcodes.

We also generated two different 10 nt length random sequences to replace the barcode of BR-I as BR-Ia and BR-Ib (Table S1) to validate the influences of barcodes to PPI signal transcoding of core sequences. We measured PdF signals of CI dimer and GFP monomer using BR-I, BR-Ia or BR-Ib as BR sequences (Fig. 3B), and found that the barcode sequences had no obvious effect on signal transcoding ability of core sequences.

To detect three specific barcode sequences simultaneously, three different beacons (Beacon-I, Beacon-II and Beacon-III, Table S1) modified by different fluorophore-quencher pairs pairs (5′-FAM/3′-Dabcy1, 5′-Cy5/3′-BHQ-2, 5′-Texas Red/3′-BHQ-2) were designed for specific detection of BR-I, BR-II and BR-III (Fig. 3C). Each beacon is a 38 nt single-stranded DNA with stem-loop secondary structure, and the loop region can recognize the specific barcode sequence.

### Quantitative properties of BAD used in PiF

**Figure 4:**
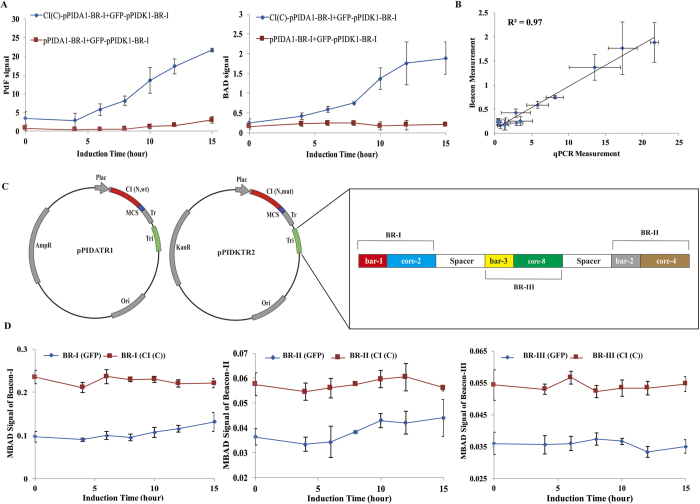
Quantitative properties of PiF. (**A**) Comparing the PdF signals of CI(N,wt)-CI(C) dimer (blue) with CI(N,wt)-GFP monomer (red) measured by qPCR, and the BAD signal of CI(N,wt)-CI(C) dimer (blue) with CI(N,wt)-GFP monomer (red) read by BAD (Error bars indicate SD of from three independent biological samples). (**B**) The linear correlation between the PdF signal measured by qPCR and the BAD signal measured by BAD (Error bars indicate SD of from three independent biological samples). (**C**) Dual-vector system for PiF assay designed in the present study. (**D**) Comparison of CI(N)-CI(C) MBAD signal of different beacons with CI(N)-GFP MBAD signal of different beacons. MBAD signals of CI(C) dimer from different beacons were all obviously stronger than GFP monomer. (Error bars indicate SD of MBAD signals from three independent biological samples).

To read out the copy numbers of BR sequences with small differences simultaneously, we chose BAD assay in this study. Because of PPI signal transcoding was quantitatively detected by qPCR as PdF signal in previous study^8^, we compared BR-I PdF signals of CI dimer and GFP monomer measured by qPCR with BR-I BAD signals of CI dimer and GFP monomer measured by Beacon-I (Fig. 4A). The result showed that there was a linear correlation between the BAD- and qPCR-measurements (Fig. 4B), indicating that both assays are interchangeable in PPI signal transcoding measurement.

The fluorescence-density changes of different beacons in the reaction were measured simultaneously by LightCycler^®^96 Real-Time PCR System (Fig. S1A). In this study, each beacon time-series reading normally has three different phases: initiate phase (I), linear phase (II) and non-linear phase (Fig. S1B), and the fluorescence change in linear phase (Phase II) of the target DNA detected by a beacon is defined as MBAD signal. The copy number of a target DNA sequence is represented by its MBAD signal (Fig. S1B), which has a linear correlation with the amount of DNA target (Fig. S1C).

### PiF can quantitatively distinguish protein dimers from monomers by measuring MBAD signal

A dual-vector system was designed for PiF, in which each vector has the same configuration but differs in antibiotic resistance, and the BR sequence and spacer sequences are linked together as a triple detection sequence (Fig. 4C). To test whether the interference of the protein monomer-BR binding signal could be ignored from the MBAD signal of dimerization, we used the C-terminal domain of CI (CI(C)) to construct CI(C)-pPIDATR1 + CI(C)-pPIDKTR2 as a positive control and GFP-pPIDATR1 + GFP-pPIDKTR2 as a negative control. Comparing the time-series MBAD signals of CI(C) dimer and GFP monomer from each beacon, we found that MBAD signals of CI(C) dimer from different beacons were all obviously stronger than GFP monomer (Fig. 4D). Using MBAD signal of GFP monomer as the reference (I_ref_), we measured the intensity of each interaction (Intensity = MBAD signal/I_ref_) with the threshold = 1.

### Simultaneously detecting multiple PPIs of different *E. coli* chemotaxis protein combinations

**Figure 5:**
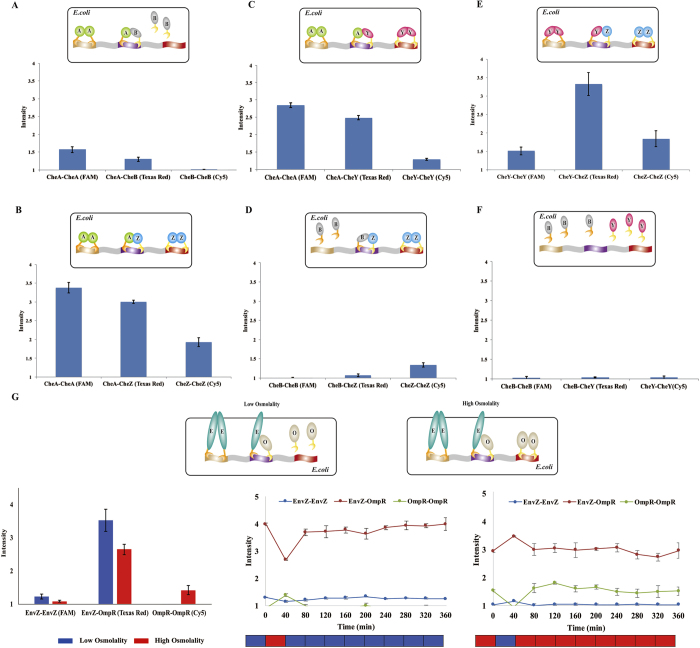
Quantitatively and simultaneously detecting multiple PPIs in one cell by PiF method. (**A**~**F**) Quantitative and simultaneous detection of three different PPIs of two *E. coli* chemotaxis proteins in one cell, including CheA (A), CheB (B), CheY(Y) and CheZ (Z). (Error bars indicate SD of interaction intensities from three independent biological samples) (G) Quantitative detection of three different PPIs of *E. coli* EnvZ (E)/OmpR (O) two-component system. Red color represents high osmolality M9 media (100 mM NaCl), blue color represents low osmolality M9 media with (8.5 mM NaCl). In the interaction dynamic changes, each color block represents the environment osmolality in 40 min. (Error bars indicate SD of interaction intensities from three independent biological samples).

Using PiF method, we simultaneously detected three different interactions of all possible binary combinations in a two-component system of *E. coli* chemotaxis^12^ under the same environmental condition (Fig. 5A–F). The qualitative results of these chemotaxis PPIs detected by PiF were similar to previous works^8^,^13^. Importantly, unlike other PPI detection methods, PiF could detect interaction intensity changes of a physical PPI due to the influence of other PPIs. The results showed that the CheA-dimerization intensity of Fig. 5A was different from that of Fig. 5B and C, suggesting that the CheA-dimerization intensity could be effected by other PPIs. The similar phenomena were also observed on the CheZ-dimerization intensity (comparing Fig. 5B–D and E) and CheY-dimerization intensity (comparing Fig. 5C–E and F). Therefore, using PiF method, we show that the intensities of physical PPIs among these proteins could affect each other for the first time.

### Simultaneously detecting dynamic changes of multiple PPIs in EnvZ/OmpR system

We further analyzed the interactions in *E. coli* osmolality sensing system to demonstrate PiF’s ability of simultaneously monitoring multiple dynamic PPIs in one cell. In this system, OmpR is activated as dimer by phosphorylation of EnvZ dimer in high osmolality environment, which reduces the interaction intensity between them^14^. Using the PiF method, we simultaneously measured three different interactions of EnvZ and OmpR in the same cell under different osmolality environments (Fig. 5G), which were consistent with previous study^14^. We also observed these interactions change with the environmental stimulations (Fig. 5G), and identified the positive correlations between EnvZ homo-dimerization and EnvZ-OmpR hetero-dimerization as well as the negative correlations between OmpR homo-dimerization and EnvZ-OmpR hetero-dimerization.

### The correlations of different PPIs and PPI correlation network

**Figure 6:**
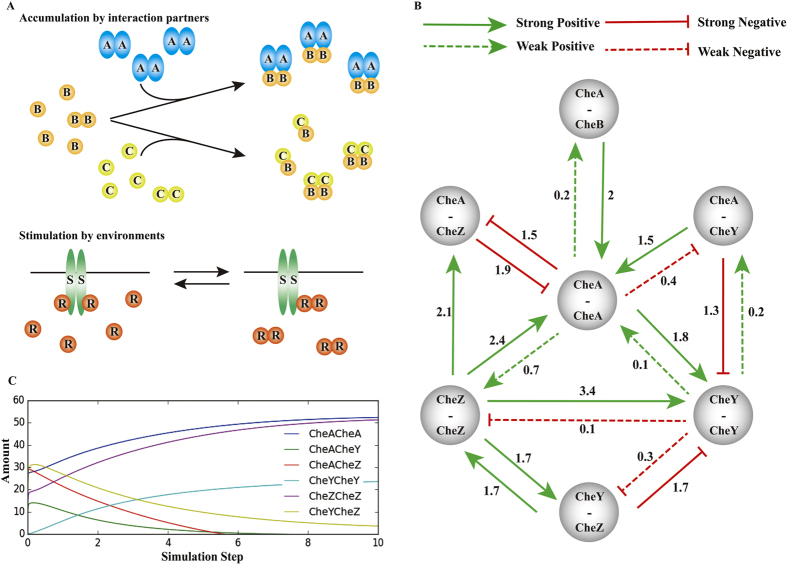
The correlations of physical PPIs in the interactome. (**A**) Two kinds of physical PPI correlations, including accumulation of different interaction partners and environment stimulated interaction intensity changes. (**B**) PPI correlation network (PPICN) of *E. coli* chemotaxis proteins. In this network, each vertex represents a physical PPI, and each directed edge connecting two vertices represents the positive or negative correlation of two PPIs. (**C**) Model simulation of chemotaxis protein dimer concentration changes. The simulation results showed there were two different dynamic patterns in this network.

These results imply two kinds of physical PPI correlations (Fig. 6A). The first is that the interaction intensity changes rely on the accumulation of different interaction partners such as the interactions of *E. coli* chemotaxis proteins (Fig. 5A–F). The second is that the interaction intensity changes are stimulated by different environments such as the interactions of *E. coli* osmolality sensing proteins (Fig. 5G). To further quantify the physical PPI correlations, we used the modular response analysis method^15^ to generate an interaction map between different physical PPIs of *E. coli* chemotaxis proteins. To make systematic perturbation, we generated a number of protein mutations to interrupt these PPIs one at a time, and then simultaneously measured their intensities in a cell by PiF (Fig. S2). We calculated the global response matrix of physical PPIs (Supplementary Information) and drew the physical PPI correlation network (PPICN) of *E. coli* chemotaxis proteins (Fig. 6B). In this network, each vertex represents a physical PPI, and each directed edge connecting two vertices represents the positive or negative correlation of two PPIs. In PPICN, most of two directed edges between two vertexes include one strong edge and one weak edge (Fig. 6B), suggesting promotional or inhibitory mutual correlations between two physical PPIs.

## Discussion

The purpose that we design PiF is to reveal the system properties of protein interactome by measuring different PPIs simultaneously. PiF method provide several advantages to protein interactome study. First, PiF can extend its capability to simultaneously and quantitatively detect multiple PPIs among more than two proteins in one cell once we find enough orthogonal pairs of DNA-binding domains and corresponding specific DNA sequences for detection. Secondly, PiF can reveal the dynamic changes of the physical PPI intensities under influences of other interaction partners or environments. Thirdly, PiF can analyze the correlations between the binary interaction pairs in a protein interactome.

As a totally new concept of protein interactome, PPICN was in agreement with previous observations. For example, CheZ dimer with a modest change of conformation and activation upon binding to phospho-CheY resulted in an increased affinity to CheA^13^,^16^; and the CheY dimerization might depend on coming into proximity when interacting with CheA dimer and CheZ dimer^13^. Furthermore, we simulated the concentrations of different dimers in time-series with their dynamic changes of the interaction intensities based on PPICN (Fig. 6C). The simulation results showed there were two different dynamic patterns in this network. First, CheA and CheZ homodimers increased rapidly to a steady high concentration state with time, which is consistent with the fact that CheA and CheZ are strong intradimers^13^, while CheY homodimer increased slowly to a steady low concentration state, which is consistent with the weak dimerization of CheY^13^. Secondly, the heterodimers of CheA, CheY and CheZ increased in a narrow time window, and then decayed with time, indicating that CheY and CheZ are released from the complex after rapid activation or deactivation process of heterodimers^13^,^17^. These results suggest that PPICN may also reveal the intrinsic properties of complex assembly and disassembly within a protein network by giving the correlation of different physical PPIs.

In this study, we demonstrated the PiF method in bacterial cells which do not have nucleus and in which all the proteins can perform PPI-driven DNA binding processes with the detection sequences inserted in expression vectors. In contrast, existence of nucleus in eukaryotic cells isolates the detection sequences located in the expression vectors from PPIs in cytoplasm. To expand the PiF technology for interactome study in eukaryotic cells, detection sequences could be separated from expression vectors as independent exogenous DNAs, and delivered directly into nucleus or cytoplasm^18^ for PiF detection. Furthermore, we simultaneously measured three different PPIs formed by two proteins, which can cover, for example, most bacterial signal transduction systems (two-component systems)^19^. However, in eukaryotic cell, many systems, such as kinase cascade system^19^, are complex cascades with more than two proteins. In these cases, many more specific DNA binding domain-DNA sequence pairs need to be designed for simultaneously measuring of different PPIs in eukaryotic interactome. The larger the interactome scale we study, the harder the design of different orthogonal pairs of DNA binding domains and specific DNA sequences. So further study is required to optimize the design and selection processes based on a proper scale of protein interactome.

## Material and Methods

### Plasmid construction

Plasmid pPIDATR1 was constructed using the pSP73 (Promega, P2221) vector as the backbone and inserting the P_lac_ promoter, the N-terminal DNA binding domain of the CI gene (CI(N,wt), cloned from lambda DNA (Thermo, SD0011)), a multiple cloning site (MCS), a terminator and the triple detection sequence (Tri) according to the BioBrick assembly standard. Plasmid pPIDKTR2 was constructed using the pSB1K3 (Biobrick) vector as the backbone and inserting the P_lac_ promoter, the N-terminal CI DNA binding domain gene mutation (CI(N,mut))), a multiple cloning site (MCS), a terminator and the triple detection sequence (Tri) according to the BioBrick assembly standard. These two plasmids were used for the PdF assay.

The *cheA, cheB, cheZ, cheY, envZ, ompR* genes were cloned from the genome of *E. coli* strain DH5α. *GFP*_*mut3b*_ was cloned from GFP_mut3b_-pSB1A2 (BioBrick, BBa_E0040). These target genes were inserted into the multiple cloning sites (MCS) of the above plasmids by homologous recombination using the In-Fusion HD Cloning Kit (Clontech, 638909).

### The PiF assay

#### *In vivo* protein expression

X-pPIDATR1 and Y-pPIDKTR2 (where X and Y are target proteins) containing the triple detection sequence (Tru) were co-transformed into *E. coli* JM109 competent cells and spread on LB agar plates containing Ampicillin (50 ng/μl) and Kanamycin (50 ng/μl) at 30 °C overnight. A single colony was diluted in 2 ml M9 His DO medium containing Ampicillin (50 ng/μl) and Kanamycin (50 ng/μl) and was shaken at 250 rpm at 37 °C overnight. An overnight culture of transformed JM109 was diluted to 2 ml in fresh M9 His DO medium containing Ampicillin (50 ng/μl), Kanamycin (50 ng/μl) and IPTG (10 μM) to reach an OD_600_ of 0.03, then shaken at 250 rpm at 37 °C for 10 hours.

#### *In vivo* protein–DNA complex extraction

After an 8-hour IPTG induction, 1 ml of cell culture was moved into a 15 ml conical centrifuge tube, and 27 μl of 37% formaldehyde was added to obtain a final concentration of 1% formaldehyde for crosslinking. After incubation at 300 rpm and 25 °C for 8 minutes, 10X glycine solution was added into this mixed culture at a final concentration of 1X glycine for crosslinking termination by incubating at 300 rpm and 25 °C for 5 minutes. The cells were then collected by centrifugation at 12,000 rpm for 4 minutes and washed twice with 1 ml of ice-cold 1X PBS. The cells were resuspended in 1 ml 1X PBS with 10 μl Halt Protease and Phosphatase Inhibitor Cocktail (Pierce 78443) and centrifuged at 1,2000 rpm for 4 minutes. The cross-linked cells were resuspended in 200 μl of Membrane Extraction Buffer (Pierce Chromatin Prep Module, 26158) containing 2 μl Halt Protease and Phosphatase Inhibitor Cocktail and incubated on ice for 10 minutes. The genophore was then collected by centrifugation at 9,000 rpm for 3 minutes. The genophore was resuspended in 200 μl MNase Digestion Buffer (Pierce Chromatin Prep Module, 26158) with 0.2 μl 1 M DTT solution, and 100 μl of the resuspended genophore was transferred into a new tube in parallel. Then, 4 μl of Micrococcal Nuclease Mix (containing 0.4 μl of 10 U/μl Micrococcal Nuclease (Pierce Chromatin Prep Module, 26158) with 3.6 μl of MNase Digestion Buffer) was added into each tube containing 100 μl resuspended genophore and incubated at 37 °C for 15 minutes for fragmentation. Next, 20 μl MNase Stop Solution (Pierce Chromatin Prep Module, 26158) was added to stop the reaction, and the genophore solution was incubated on ice for 5 minutes. The genophore was collected by centrifugation at 9,000 rpm for 5 minutes. The genophore was resuspended in 50 μl Nuclear Extraction Buffer (Pierce Chromatin Prep Module, 26158) and incubated on ice for 15 minutes. The genophore was extracted by centrifugation at 9,000 rpm for 5 minutes, and the extracted solution was transferred to a new tube.

#### *In vitro* DNase I digestion and purification

For digestion, 2 μl DNase I (2,000 U/mL, NEB, M0303S) was added into 50 μl extracted solution and incubated at 37 °C overnight. The reaction was stopped by incubating at 75 °C for 10 minutes. Next, a solution containing 6.6 μl of nuclease free water, 2.4 μl 5 M NaCl and 1 μl proteinase K (20 mg/ml) was added to the digested genophore solution and incubated at 65 °C for 90 minutes to remove proteins from the protein-DNA complexes. Then DNA was purified by using the UNIQ-10 Spin Column Oligo DNA Purification Kit (Sangon Biotech, SK1143).

#### Quantification

Concentrations of different target DNA sequences were measured simultaneously by using beacon-assisted detection method.

### Beacon-assisted detection of target DNA

#### Molecular beacon preparation

There three different Beacons used in this study (Beacon-I for BR-I, Beacon-II for BR-II and Beacon-III for BR-III), which are modified by different fluorophore-quencher pairs (5’-FAM/3’-Dabcy1, 5’-Cy5/3’-BHQ-2, 5’-Texas Red/3’-BHQ-2). These modified beacons are synthesized and purified by *Sangon* Biotech, and supplied as DNA pellet. 80 μM solution of the beacon is prepared in molecular beacon buffer (20 mM Tris and 1 mM MgCl_2_, PH = 8.0) and incubated at 95 °C for 5 min, then it is allowed to slowly cool to room temperature over 30 min. The beacon solution is stored at −20 °C.

#### Experiment procedure

To detect the target DNA sequence, prepare solution A and B listed in Table S2. Then incubate solution B at 95 °C for 5 min and cool it on ice immediately. Aliquot solution B into 8-tube strip (Roche, 06612601001) placed on ice, then initiate the reaction by adding solution A to solution B and mix well. Place the 8-tube strip into LightCycler^®^96 Real-Time PCR System (Roche) and start the incubation program. In the incubation program, there are three detection formats been chosen, including FAM, Texas Red and Cy5, and thermal cycles are 40 cycles (40 °C, 30 s).

#### Data analyzing

From LightCycler^®^96 Real-Time PCR System, get the fluorescence readings of three beacons at the same time. Each beacon time-series reading normally has three different phases, the fluorescence change in linear phase of the target DNA detected by a beacon is defined as MBAD signal.

### Protein-protein interaction correlation network drawing

#### Modular response analysis

An interaction map of different protein-protein interactions can be discovered by using mutations (CheA (L310S)^20^, CheA (L126A)^21^, CheA (F214A)^22^,^23^, CheZ (L110P)^24^, CheZ (D143G)^24^, CheY (D12E)^25^) to interrupt interactions in this study and measuring global response only. In our study, three interactions are measured simultaneously. There are three major step in protein-protein interaction correlation network drawing: first, use a perturbation that affects a single interaction and measure the difference in the steady-state levels of interaction intensity before and after the perturbation; then, according to Eq. 1, calculate the global fraction changes of three interactions and repeat for remaining interactions to calculate global response matrix **R**_**p**_ (Equation 2); finally, apply matrix **R**_**p**_in Eq. 3 to get network interaction map **r**, which is the protein-protein interaction correlation network of these three interactions.

***Equations.*** Global fraction change


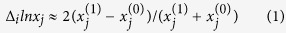


where 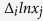 is the global fraction change of interaction j under the perturbation of interaction i, 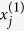 is the interaction intensity after perturbation and 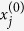 is the interaction intensity before perturbation.

Global response matrix


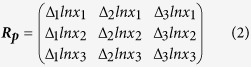


Network interaction map


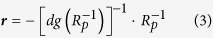


### Dynamic simulation of protein-protein interaction correlation network

According to protein-protein interaction correlation network of *E. coli* chemotaxis, there are seven different interactions to form seven different protein complexes:


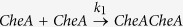



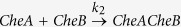



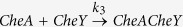



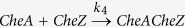



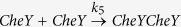



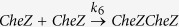



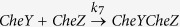


where k_1_, k_2_, k_3_, k_4_, k_5_, k_6_ and k_7_ are the interaction intensities of these interactions.

Define the concentration of each dimer as x_1_ (CheACheA), x_2_ (CheACheB), x_3_ (CheACheY), x_4_ (CheACheZ), x_5_ (CheYCheY), x_6_ (CheZCheZ), x_7_ (CheYCheZ) and set d_1_, d_2_, d_3_, d_4_, d_5_, d_6_ and d_7_ as dissociate rates. The total concentrations^26^ of CheA, CheB, CheY and CheZ are C_a_, C_b_, C_y_ and C_z_, then we have following 14 ordinary differential equations based on the protein-protein interaction correlation network (PPICN):


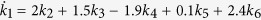



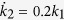



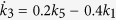



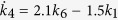



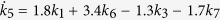



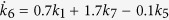



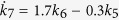



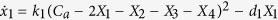



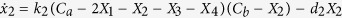



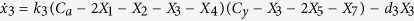



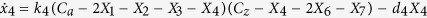



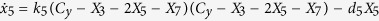



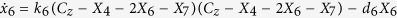



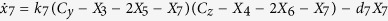


According to these ordinary differential equations, we calculated each PPI intensity and concentration of each dimer in time-series by using following parameters shown in Table S3.

## Additional Information

**How to cite this article:** Luo, S.-W. *et al*. Simultaneously measuring multiple protein interactions and their correlations in a cell by Protein-interactome Footprinting. *Sci. Rep.* **7**, 45169; doi: 10.1038/srep45169 (2017).

**Publisher's note:** Springer Nature remains neutral with regard to jurisdictional claims in published maps and institutional affiliations.

## Supplementary Material

Supplementary Information
