# Supplementary material for: Simultaneously measuring multiple protein interactions and their correlations in a cell by Protein-interactome Footprinting
Source: Sci Rep. 2017 Mar 24;7:45169. doi: 10.1038/srep45169 (PMC5364535; doi:10.1038/srep45169)
Supplement: Supplementary Information [file srep45169-s1.pdf]

Simultaneously measuring multiple protein interactions and their correlations in a cell by Protein-interactome Footprinting

Si-Wei Luo[1,2], Zhi Liang[2*] & Jia-Rui Wu[1,2,3*]

[1]Key Laboratory of Systems Biology, Institute of Biochemistry and Cell Biology, Shanghai Institutes for Biological Sciences, Chinese Academy of Sciences, Shanghai, China

[2]Hefei National Laboratory for Physical Sciences at Microscale and School of Life Sciences, University of Science & Technology of China, Hefei, China

[3]School of Life Science and Technology, ShanghaiTech University, Shanghai, China

*Correspondence to: Z.L., E-mail: liangzhi@ustc.edu.cn; J.R.W., E-mail: wujr@sibs.ac.cn.

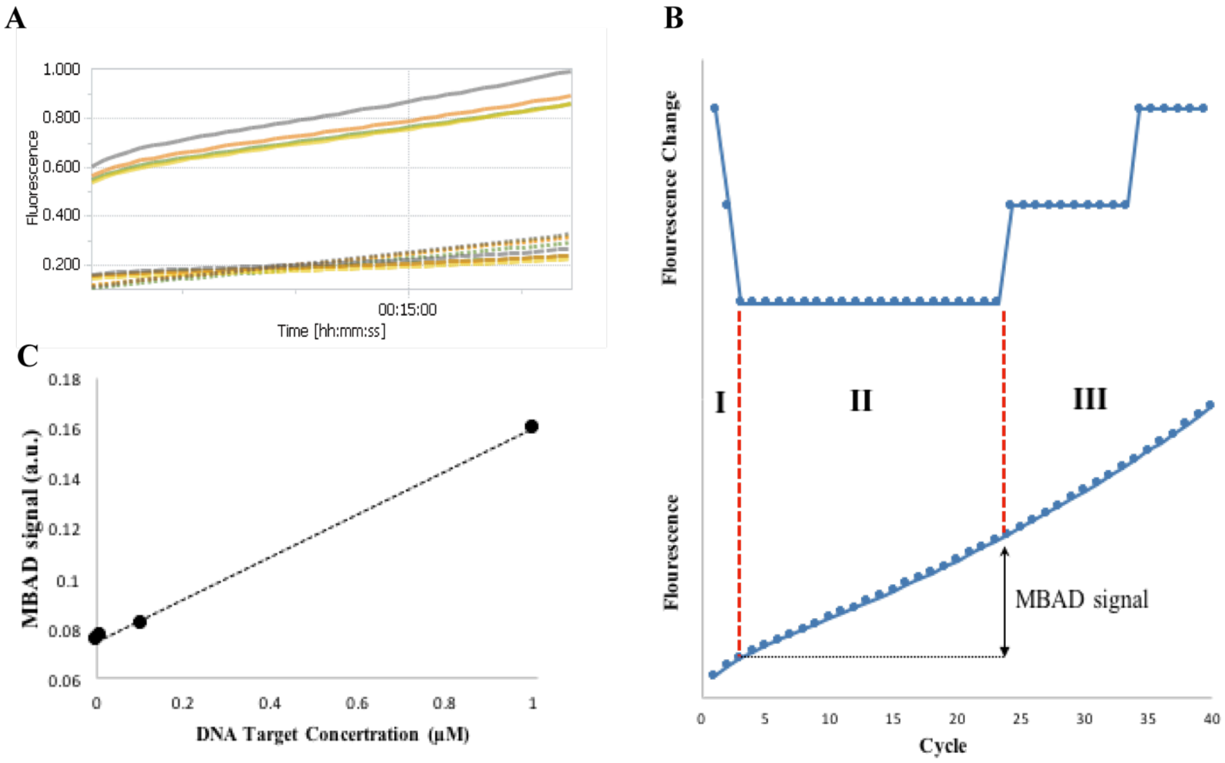

Fig. S1 (A) The output of BAD fluorescence readings in LightCycler®96 Real-Time PCR System. Different forms of lines represent the readings of different beacons. **(B)** Each beacon time-series reading normally has three different phases: initiate phase (I), linear phase (II) and non-linear phase. In some experiments, there are only two phases: I+II or II+III. **(C)** The BAD reading of Beacon-I from different amount BR-I in the mix with 0.1μM BR-II and 0.1μM BR-III.

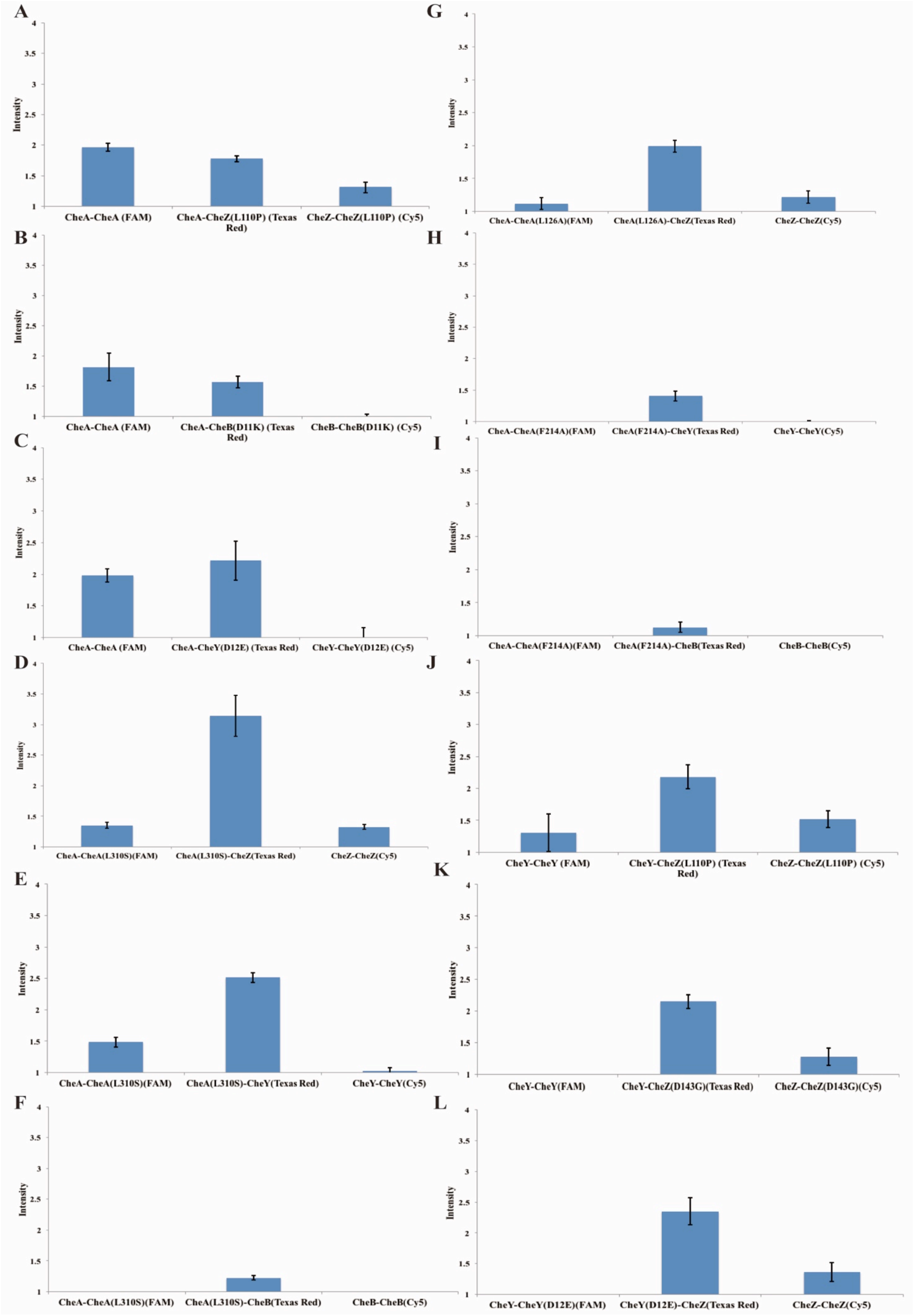
A
Intensity
CheA-CheA (FAM)
CheA-CheZ(L110P) (Texas Red)
CheZ-CheZ(L110P) (Cy5)
B
Intensity
CheA-CheA (FAM)
CheA-CheB(D11K) (Texas Red)
CheB-CheB(D11K) (Cy5)
C
Intensity
CheA-CheA (FAM)
CheA-CheY(D12E) (Texas Red)
CheY-CheY(D12E) (Cy5)
D
Intensity
CheA-CheA(L310S)(FAM)
CheA(L310S)-CheZ(Texas Red)
CheZ-CheZ(Cy5)
E
Intensity
CheA-CheA(L310S)(FAM)
CheA(L310S)-CheY(Texas Red)
CheY-CheY(Cy5)
F
Intensity
CheA-CheA(L310S)(FAM)
CheA(L310S)-CheB(Texas Red)
CheB-CheB(Cy5)
G
Intensity
CheA-CheA(L126A)(FAM)
CheA(L126A)-CheZ(Texas Red)
CheZ-CheZ(Cy5)
H
Intensity
CheA-CheA(F214A)(FAM)
CheA(F214A)-CheY(Texas Red)
CheY-CheY(Cy5)
I
Intensity
CheA-CheA(F214A)(FAM)
CheA(F214A)-CheB(Texas Red)
CheB-CheB(Cy5)
J
Intensity
CheY-CheY (FAM)
CheY-CheZ(L110P) (Texas Red)
CheZ-CheZ(L110P) (Cy5)
K
Intensity
CheY-CheY(FAM)
CheY-CheZ(D143G)(Texas Red)
CheZ-CheZ(D143G)(Cy5)
L
Intensity
CheY-CheY(D12E)(FAM)
CheY(D12E)-CheZ(Texas Red)
CheZ-CheZ(Cy5)

Fig. S2 Using PiF method measure three different interaction intensities of each binary combinations of $CheA_L$, CheB, CheY, CheZ and their mutations in one cell simultaneously**. (A)** $CheA_L$+CheZ (L110P), **(B)** $CheA_L$+CheB (D11K), **(C)** $CheA_L$+CheY (D12E), **(D)** $CheA_L$ (L310S)+CheZ, **(E)** $CheA_L$ (L310S)+CheY, **(F)** $CheA_L$ (L310S)+CheB, **(G)** $CheA_L$ (L126A)+CheZ, **(H)** $CheA_L$ (F214A)+CheY, **(I)** $CheA_L$ (F214A)+CheB, **(J)** CheY+CheZ (L110P), **(K)** CheY+CheZ (D143G), **(L)** CheY (D12E)+CheZ.

Table. S1

Core-1	GGGTGTTATCTACCTCTGGCGGTGATAACTTCATCTCTGCCCTGTGG
Core-2	GGGTGTTATCTACCTCTGGCCGTGATAACTTCATCTCTGCCCTGTGG
Core-3	GGGTGTTATCTACCTCTGGCGGTGCTAACTTCATCTCTGCCCTGTGG
Core-4	GGGTGTTATCTACCCCTGGCGGTGATAACTTCATCTCTGCCCTGTGG
Core-5	GGGTGTTATCTACCCCTGGCTGTGATAACTTCATCTCTGCCCTGTGG
Core-6	GGGTGTTATCTACCCCTGGCCGTGATAACTTCATCTCTGCCCTGTGG
Core-7	GGGTGTTATCTACCCCAGGCCGTGATAACTTCATCTCTGCCCTGTGG
Core-8	GGGTGTTATCTACCCCAGTCCGTGATAACTTCATCTCTGCCCTGTGG
bar-1*	AGCAAAATCA
bar-2*	GCCTGTCATC
bar-3*	ACGAATTTCT
BR-I*	AGCAAAATCAGGGTGTTATCTACCTCTGGCCGTGATAACTTCATCTCTGCCCTGTGG
BR-Ia*	CCTTCCTTACGGGTGTTATCTACCTCTGGCCGTGATAACTTCATCTCTGCCCTGTGG
BR-Ib*	TTGCCGCTCTGGGTGTTATCTACCTCTGGCCGTGATAACTTCATCTCTGCCCTGTGG
BR-II*	GCCTGTCATCGGGTGTTATCTACCCCTGGCGGTGATAACTTCATCTCTGCCCTGTGG
BR-III*	ACGAATTTCTGGGTGTTATCTACCCCAGTCCGTGATAACTTCATCTCTGCCCTGTGG
Beacon-I*	GCGCCGTCGAGCAAAATCAGGGTCCTCAGCGACGGCGC
Beacon-II*	GCGCCGTCGGCCTGTCATCGGGTCCTCAGCGACGGCGC
Beacon-III*	GCGCCGTCGACGAATTTCTGGGTCCTCAGCGACGGCGC
Spacer	TCGAGTAGTGTGAGGGGAGACTATAACGTAGCATCGTTCAAATTCAACAT
BAD Primer	GCCGTCGC
CI(N,wt)	ATGAGCACAAAAAAGAAACCATTAACACAAGAGCAGCTTGAGGACGCACGTCGCCTTAAAGCA<br>ATTTATGAAAAAAAGAAAAATGAACTTGGCTTATCCCAGGAATCTGTCGCAGACAAGATGGGG<br>ATGGGGCAGTCAGGCGTTGGTGCTTTATTTAATGGCATCAATGCATTAAATGCTTATAACGCCG<br>CATTGCTTACAAAAATTCTCAAAGTTAGCGTTGAAGAATTTAGCCCTTCAATCGCCAGAGAAAT<br>CTACGAGATGTATGAAGCGGTTAGTATGCAGCCGTCACTTAGAAGTGAGTATGAGTACCCTGTT<br>TTTTCACATGTTCAG
CI(N,mut)	ATGAGCACAAAAAAGAAACCATTAACACAAGAGCAGCTTGAGGACGCACGTCGCCTTAAAGCA<br>ATTTATGAAAAAAAGAAAAATGAACTTGGCTTATCCCAGGAATCTGTCGCAGACAAGATGGGG<br>ATGGGGCAGTCAGCGATTAATAAGGCATTTAATGGCATCAATGCATTAAATGCTTATAACGCCG<br>CATTGCTTACAAAAATTCTCAAAGTTAGCGTTGAAGAATTTAGCCCTTCAATCGCCAGAGAAAT<br>CTACGAGATGTATGAAGCGGTTAGTATGCAGCCGTCACTTAGAAGTGAGTATGAGTACCCTGTT<br>TTTTCACATGTTCAG

* The red sequence is the barcode sequence.

Table. S2

**Solution A**	**Volume (μl)**	**Solution B**	**Volume (μl)**
Bst DNA polymerase (NEB, M0275)	0.25	Sample for detection	1
Nb.BbvCI (NEB, R0631S)	0.25	Beacon0	0.25
BSA solution (NEB, B9001S)	0.5	Beacon8	0.25
dNTP Mix 2mM (Thermo, R0242)	1	Beacon14	0.25
NEBuffer 2 (NEB, B7002S)	2	deionized water	4.5
BAD primer	4		
deionized water	5.75		

Table.S3

Initiate k_1	2.4	Initiate x_4	0	d_3	0.5
Initiate k_2	0.2	Initiate x_5	0	d_4	0.5
Initiate k_3	1.5	Initiate x_6	0	d_5	1
Initiate k_4	2	Initiate x_7	0	d_6	0.1
Initiate k_5	0	C_a	100	d_7	0.5
Initiate k_6	0.9	C_b	2		
Initiate k_7	2.3	C_y	50		
Initiate x_1	0	C_z	100		
Initiate x_2	0	d_1	0.1		
Initiate x_3	0	d_2	0.5		
